# Supplementary material for: Optimizing a 3D convolutional neural network to detect Alzheimer’s disease based on MRI
Source: PeerJ Comput Sci. 2025 Aug 29;11:e3129. doi: 10.7717/peerj-cs.3129 (PMC12453851; doi:10.7717/peerj-cs.3129)
Supplement: Supplemental Information 2 [file peerj-cs-11-3129-s002.pdf]

Author Contribution Statement – Abdulmajeed Almuaibed

Full Name: Abdulmajeed Almuaibed

Role: Contributing Author

Reason for Addition:

The omission of Abdulmajeed Almuaibed from the original author list was an unintentional mistake. We regretfully overlooked including his name during the initial submission and sincerely apologize for this oversight. Upon revision, we have reviewed and acknowledged his substantial contributions, which clearly merit authorship.

Detailed Contributions:

Abdulmajeed Almuaibed made significant contributions to the revised version of the manuscript, including:

Designing and structuring the experimental framework.

Conducting new experiments to enhance the model's evaluation.

Analyzing and interpreting the experimental data.

Preparing high-quality figures and tables that are now part of the final submission.

Revising the manuscript content for clarity, accuracy, and consistency.

These contributions meet the authorship criteria defined by the journal and warrant his inclusion in the final author list. All co-authors have reviewed and approved this addition.

Confirmation:

This form is submitted to fulfill the journal's requirement for documentation of new author contributions. The full author team agrees with this update, and the revised manuscript reflects Abdulmajeed Almuaibed's contributions via tracked changes and comments.
